# Improving Access to Prenatal Care of High-Risk Pregnant Women in Houston, Texas: The Role of Nurse Driven Care Management

**DOI:** 10.34763/jmotherandchild.20252901.d-25-00002

**Published:** 2025-07-02

**Authors:** Mattie Mason, Abayomi Joseph Afe, Jamesia Fransaw

**Affiliations:** New Life Perinatal Health Care Services, Inc., Houston, Texas, USA; School of Health Sciences, Purdue University Global, Indianapolis, USA

**Keywords:** Prenatal, Medicaid, Risk, Pregnancy, Nurse

## Abstract

**Background:**

Prenatal care in the US is often mediated through managed care organisations. Other community-based health organisations also implement nurse-led care programs to help pregnant women navigate prenatal care services. The aim of this study is to assess the impact of such organisational services.

**Material and methods:**

This was a retrospective cohort analysis of data generated from providing community-based care management services to pregnant women in Houston, Texas. Clients' characteristics and outcomes were analysed and described.

**Results:**

About 60 pregnant women received care management services between 2022 and 2023. Out of these, 24 (40%) were teenagers (13–19 years of age), 28 (47%) were young adults (20–26 years), 5 (8%) were 27–30 years, and 3 (5%) were older than 35 years. The youngest patient was 15 years old and the oldest was 39 years. 50% (n = 30) were African-American, 38% (n = 23) were Hispanic, and 12% (n = 7) were white. 48% (n = 29) were in their second trimester, 30% (n = 18) were in their first trimester and 22% (n = 13) were in their third trimester. The earliest gestational age was four weeks, the oldest gestational age was 38 weeks, and the average was 20 weeks. The most common medical risk factors were anxiety, depression, and epilepsy. Others included anaemia, diabetes, alcoholism, smoking, PCOS, thalassemia, renal disease, COVID-19 infection, Lupus erythematosus, multiple gestation, and previous miscarriage. Half of the women, n = 27 (46%), had incorrect Medicaid health insurance that did not cover pregnancy care, and the other half, n=32 (54%), had no health insurance at all.

**Conclusion:**

While it took an average of 53 days for the women in this study to get enrolled in a managed care organisation, it only took an average of 22 days for them to attend their first doctor's appointment when care was directly coordinated by a nurse led community-based health organization. This speaks to the efficacy of nurse-led, community-based care management in improving early access to prenatal care.

## 1. Introduction

“Prenatal care” refers to the health care women receive during pregnancy. For maximum effectiveness, this care must be started early and consist of regular visits with a health care provider [1]. Prenatal care promotes a healthy pregnancy, a healthy mother and a healthy foetus. Women who do not access prenatal care are three times as likely to deliver a low-birth-weight infant and have an increased risk of infant death [2].

The goals of prenatal care can be categorised into medical care, including screening for and management of chronic conditions and pregnancy complications; anticipatory guidance for pregnancy, birth, postpartum period, and parenting; and psychosocial support, such as management of mental health and nonmedical factors that may affect pregnant women's access to care and their achievement of healthy outcomes [3].

Prenatal care service is a proven strategy for reducing maternal and infant mortality [4]. The United States has the highest maternal death rate of any high-income nation, with a mortality rate of 22 per 100,000 live births in 2022. This is more than double or triple the rate seen in most other high-income countries [5]. Within the U.S., the rate varies by state and socioeconomic factors like race; the rate is lowest for Asian-American women and highest for Black women. Over 80 percent of maternal deaths are likely preventable [6]. However, the number of maternal deaths in 2022 was lower than in previous years.

Nurses, especially midwives, contribute immensely to the improved quality and experience of care for women. They constitute an essential component of the reproductive and maternal health system. However, they are not systematically incorporated into the provision of essential maternity care services in the US. Some U.S. states have strengthened access to midwives – leading to improved outcomes – but midwifery services are not uniformly covered by private insurance plans across the country [5].

### 1.1. Texas Targeted case management for high-risk pregnant women and infants

The Texas Targeted case management services for pregnant women and infants program defines case management as a program that assesses a family's overall needs, including those of pregnant women and their infants, and develops and implements plans to meet those needs, which may be medical, social, developmental, nutritional, educational and/or spiritual.

These services are provided by privately-owned health firms known as case management organisations (CMOs). Case managers are the team leaders who coordinate case management services in such organisations. Case managers may be registered nurses or certified social workers licensed to practice in Texas. The main objective of case management services is to assist eligible individuals in their access to and utilisation of necessary medical, social, educational, development and other health services. Case managers set up a series of meetings with their clients, starting with the initial intake or admission meeting, during which assessment of needs and risk status is carried out. Clients are given the option to accept or decline case-management services. For those that accept, a service plan is developed in order to address the identified needs and risks, according to priority; set up measurable goals and objectives; and outline the responsibilities of all parties concerned, including the case manager, the client/family, and other stakeholders.

Follow-up meetings are then held with the prenatal clients every month before delivery, and at five and 30 days postpartum. Newborns who are high-risk are also seen by case managers within the first two weeks of life and monthly throughout their first year. Low-risk infants are seen at the ages of two, four, six, nine and 12 months.

### 1.2. Nurse-led care management of pregnant women

Because of the differences in training and education between nurses and social workers, case-management services provided or led by nurses are expected to have different outcomes compared to those led by other health professionals. Several studies have shown that nurses play vital roles in case management [7]. Naturally, central to most of nurses' roles are functions such as care coordination; development of care plans based on individuals' needs and preferences; health education and counselling for patients and families within care settings and during discharge; and facilitating continuity of care for people across settings and providers [8]. Care coordination, which constitutes the core of case management, has long been a core professional standard and competency for registered nurses [8]. Care coordination is effective in overcoming obstacles presented by health care system, such as fragmentation, communication, billing/costs, and poor access to quality care [9]. Several nurse-driven care management programs, such as the national recognised Camden Core Mode for people with complex medical and social needs, have been shown to improve patient health and satisfaction and control health care costs [10,11,12].

### 1.3. Research goal

To demonstrate the impact of nurse-driven care management in facilitating access to prenatal care for high-risk pregnant women.

### 1.4. Objectives

1. To determine the demographics of women enrolled in care management for prenatal care
2. To determine common risks among pregnant women enrolled in care management for prenatal care
3. To determine the time interval between admission and enrolment in managed care
4. To determine the interval period between admission and first doctor visit
5. To determine delivery outcomes among the registered clients
6. To make recommendations concerning this process

## 2. Materials and methods

### 2.1. Study design

This is a retrospective cohort analysis of data generated from providing care coordination to high-risk pregnant clients in the care of a community-based health organisation.

### 2.2. Study participants

A total of 60 pregnant women who were admitted into care within the city of Houston.

### 2.3. Study duration

The women were admitted into care between 2022 and 2023.

### 2.4. Community-based health care coordination

#### 2.4.1. Community health organisations

A community health organisation known as the New Life Perinatal Health Care Services, Inc. founded in 1992 provides targeted case management services for mothers and babies between 1992 and 2002. The service was renamed to Case Management for Children and Pregnant Women (CPW) in 2003.

The organisation is dedicated to advocating for and advancing the quality of health care experience/life skills for mothers and babies, empowering parents/families to be healthy and self-sufficient. The organisation strategy entails the use of a family-centred, nurse led care management to provide health care services to pregnant women and their babies.

#### 2.4.2. Care coordination services

All 60 clients were assessed at admission to determine their insurance/Medicaid status, and identify their risk factors and individual needs as well as any gaps in their care. Based on this, individualised health care plans were developed and implemented until all needs were met and clients were enrolled with a Managed Care Organisation (MCO).

Clients with high-risk conditions were scheduled and followed up with for their first prenatal care visits without Medicaid or with a Medicaid plan that did not support pregnancy care. Other services provided by New Life prenatal care include the following:

1. Pairing case managers with clients based upon the client's special needs and zip codes.
2. Home/virtual visits
3. Getting doctors' appointments
4. Enrolment in appropriate Medicaid services
5. Accessing community resources such as nutrition, mental health, transportation, education.

### 2.5. Data analysis

A descriptive statistical analysis using frequency distribution was used to analyse demographic data, such as maternal age, gestational age, race, and pregnancy-associated risks. Primary outcomes of interest included the time interval between admission into care and enrolment by case management organisation; the time interval between admission into care and first doctor's visit; and pregnancy outcomes. Inferential statistics using bivariate analysis were used to determine the association between maternal demographics and pregnancy risks.

## 3. Results

### 3.1. Data analysis

#### 3.1.1. Maternal age at admission

Of the 60 pregnant women who enrolled in managed care, 24 (40%) were teenagers (13–19 years), 28 (47%) were young adults (20–26 years), followed by five (8%) in the 27–30 year age bracket and three and elderly or geriatric mothers (older than 35 years) (5%). The youngest patient was 15 years old and the oldest was 39 years; the average age was 22 years and the modal or commonest age was 18 years (Table 1).

**Table 1.** Maternal age of enrolled clients

**Age Group**	**Count**	**% Frequency**
13–19 (Teenage)	24	40%
20–26 (Young adults)	28	47%
27–33	5	8%
35–40 (Geriatric)	3	5%

**Grand Total**	**60**	**100%**

#### 3.1.2. Maternal race

According to Table 2, 50% (n = 30) of the enrolled women were African American, followed by Hispanic women, accounting for 38% (n = 23) of the sample. Women who identified as white made up 12% (n = 7) of the enrolled sample.

**Table 2.** Race distribution of pregnant clients

**Race**	**Count**	**% Frequency**
African-American	30	50%
Hispanic	23	38%
White	7	12%

**Grand Total**	**60**	**100%**

#### 3.1.3. Gestational age at admission into care

At the point of admission into care, about 48% of the women (n = 29) were in their second trimester, 30% (n = 18) were in their first trimester and 22% (n=13) were in the late third trimester. The earliest gestational age was four weeks and the oldest gestational age was 38 weeks (Table 3). The average gestational age was 20 weeks and the commonest or modal gestational age was 13.5 weeks.

**Table 3.** Gestation weeks at admission

**Trimester**	**Count**	**% Frequency**
First trimester (1–12 weeks)	18	30%
Second trimester (13–26 weeks)	29	48%
Third trimester (27–40 weeks)	13	22%

**Grand Total**	**60**	**100%**

#### 3.1.4. Pregnancy-associated risks

The common risks identified among the women are grouped into Medicaid risk/Insurance, medical complication risks, prenatal risks and gestational risks (Table 4). Medicaid risks include not having Medicaid health insurance at all or having Medicaid that does not cover pregnancy care. Almost half of the women, n = 27 (46%), had incorrect wrong Medicaid coverage that did not cover pregnancy care, and slightly above half, n = 32 (54%), had no Medicaid coverage at all. Pregnancy Medicaid is the insurance that covers both prenatal care and delivery. Prenatal risks include late registration for prenatal found in 32(52%) of the women and a case of maternal disability. The commonest medical complications and risks were anxiety, at n = 3 (5%), depression, at n = 5 (8%), and seizure disorders, at n = 2 (3%). Other medical complications included anaemia (n = 1), diabetes (n = 1), alcoholism (n = 1), smoking (n = 1) PCOS (n=1), thalassemia (n = 1), renal disease (n = 1), COVID-19 infection (n = 1) and lupus erythematosus (n=1). In the category of gestational risks, teenage pregnancy accounted for n = 24 (40%), while advanced maternal age accounted for 3% (n = 5). Two women had multiple gestation (3%), and one (2%) had a history of miscarriage in a previous pregnancy.

**Table 4.** Identified pregnancy-associated risks

**Medicaid Risk**	**Count**	**% Frequency**
No Medicaid	32	53%
Wrong Medicaid	27	45%
Correct Medicaid	1	2%

**Grand total**	**60**	**100%**

**Prenatal Risk**	**Count**	**% Frequency**
Late prenatal registration	32	53%
Disability	1	2%
No prenatal risks	27	45%

**Grand total**	**60**	**100%**

**Gestational Risk**	**Count**	**% Frequency**
Advanced maternal age	3	5%
History of miscarriage	1	2%
Multiple gestation	2	3%
Teenage pregnancy	24	40%
No gestational risk	30	50%

**Grand total**	**60**	**100%**

**Medical Complications**	**Count**	**% Frequency**
Smoking	1	2%
Alcoholism	1	2%
Anaemia	1	2%
Anxiety	3	5%
Depression	5	8%
Diabetes	1	2%
Failed tubal ligation	1	2%
Kidney problems	1	2%
Lupus erythematosus	1	2%
COVID-19	1	2%
PCOS	1	2%
Schizophrenia	1	2%
Seizure disorder	2	3%
Thalassemia	1	2%
No medical complications	39	65%

**Grand total**	**60**	**100%**

#### 3.1.5. Pregnancy risk distribution based on maternal race

As seen in Table 5, women of African-American descent accounted for half of the population with Medicaid health-insurance risk (51%) and at risk of medical complications (53%), followed by Hispanic women, who accounted for 22 (37%) of those with Medicaid health insurance risk and 6 (32%) of those at risk of medical complications. Hispanic women constituted half of the women with gestational age-related risks (50%) and risks associated with late prenatal registration (52%). White women were the least-represented group in terms of Medicaid insurance risk, at n = 7 (12%), medical complication risk, at n= 3 (16%), gestational risk, at n = 2 (20%), and prenatal risk, at n = 1 (3%).

**Table 5.** TRisk Distribution among Races

**Race**	**Medicaid Risk**	**Medical Complication Risk**	**Gestational age Risk**	**Prenatal Registration Risk**
African American	30(51%)	10(53%)	3(30%)	15(45%)
Hispanic	22(37%)	6(32%)	5(50%)	17(52%)
White	7(12%)	3(16%)	2(20%)	1(3%)

**Grand Total**	**59(100%)**	**19(100%)**	**10(100%)**	**33(100%)**

#### 3.1.6. Interval between admission and enrolment by managed-care organisation

**Figure 1.**
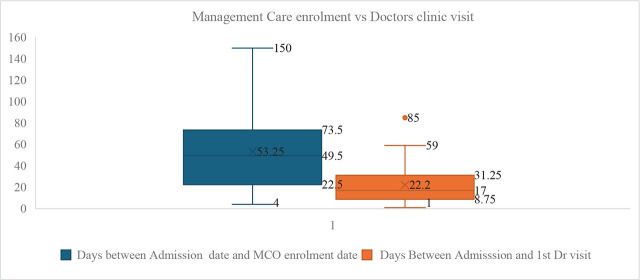
Care Management enrolment vs Doctors clinic visit.

It could take anywhere from four days to 150 days (3 months) for a newly admitted client to be enrolled by managed care organisation (Fig. 1), and the average interval was 53 days.

#### 3.1.7. Interval between admission into care and first doctor's visit

The average time interval between admission and first doctor's visit ranged from one day to 59 days, with an average of 22 days. There was an isolated case where there were 85 days before the first doctor's visit (Figure 1).

#### 3.1.8. Pregnancy outcomes

**Figure 2.**
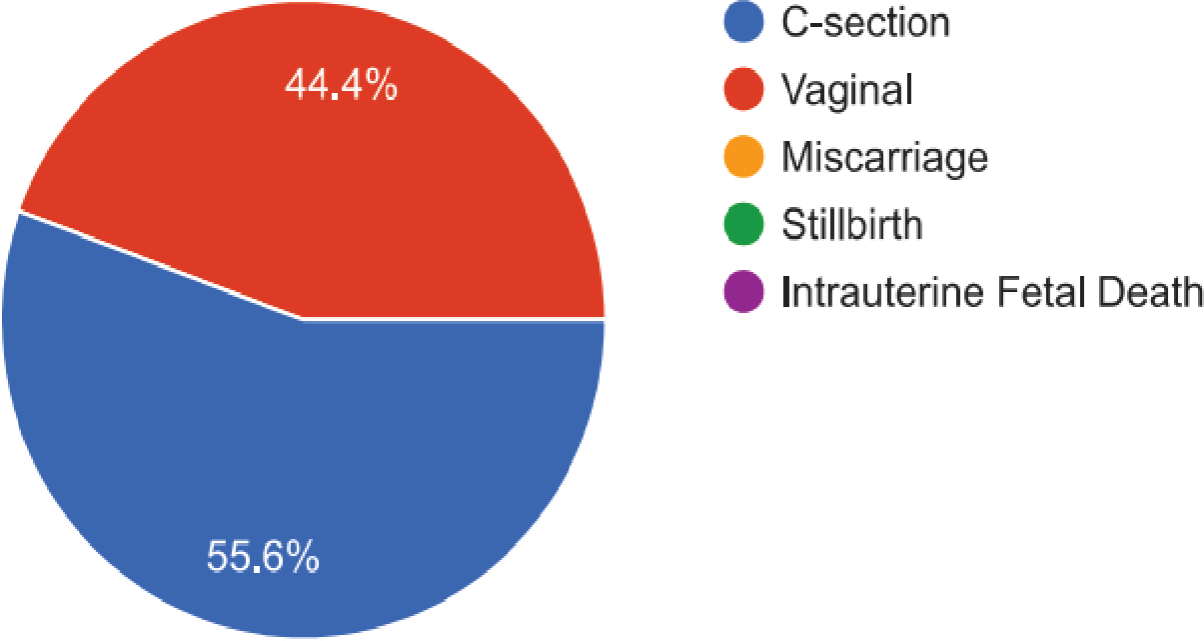
Delivery Outcomes.

**Figure 3.**
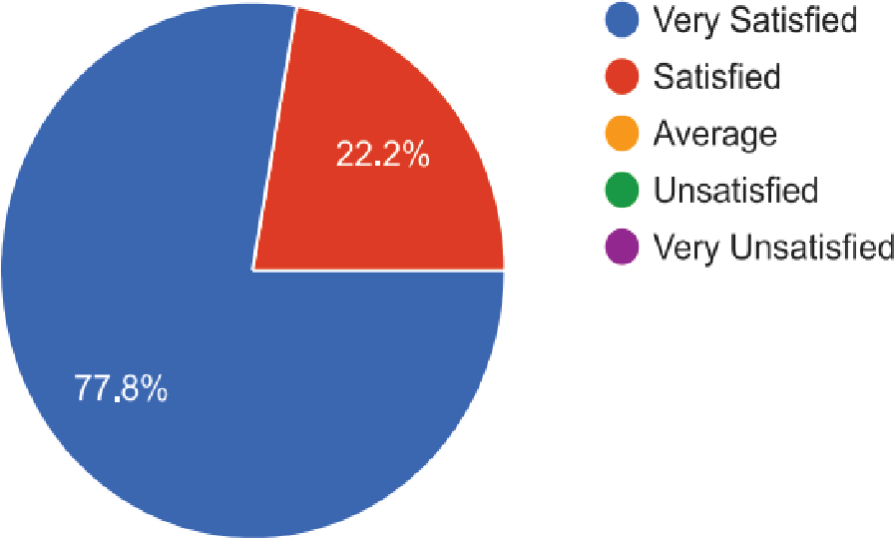
Satisfaction with the level of prenatal service.

All the 60 women delivered live babies, with 56% delivering vaginally and 44% delivering via caesarean section based on indications (Figure 2). They all expressed satisfaction with the nurse led prenatal enrolment services, ranging from 77.8% very satisfied and 22.2% moderately satisfied (Figure 3).

## 4. Discussion

The high proportion of young adults (47%) and teenagers (40%) among the study cohort showed that most women start child-bearing early in their reproductive years. This was further supported by the fact that the youngest woman was 15 years old and the oldest was 39 years old at the point of admission. These age extremes are associated with risks to both mothers and babies [13]. A similar nationwide study [14] showed that 82.5% of pregnant women in the US were 18–34 years of age.

About 50% of the pregnant women were African-American, 38% were Hispanic and 12% were white. This is a reverse of the demographic distribution within the City of Houston, the population of which is 44.83% Hispanic and 55.17% non-Hispanic, of which 42.77% are white and 39.89% are African-American [15]. African-American women constituted the highest proportion in this study because of the study population, which is made of women with high-risk pregnancies. This is in line with findings from other studies that showed that African-American women are three to four times more likely to die from pregnancy-related complications than white women [16]. Some of the reasons for this disparity include racism, lack of access to care, and poor quality of care [17].

About half (48%) of the women were in their second trimester, 30% were in their first trimester and 22% were in third trimester at the point of being admitted into prenatal care. This reflects a common delay in commencement of prenatal care among high-risk pregnant women, with about 70% delaying prenatal care registration until the second or third trimester. This picture is different from what is seen in the general US population; among women who gave birth in 2016, about 77.1% initiated prenatal care in the first trimester of pregnancy, 4.6% did so in the third trimester, and 1.6% of women received no care at all [18]. Other studies that have demonstrated similar delays in commencement of prenatal care. Some of the reasons for these delays include women not being aware they were pregnant; structural or financial barriers in the health care system; lack of available doctors' appointments; not having money or insurance to pay for the visit; the patient's doctor or health plan being unwilling to start care until later in pregnancy; and the patient not having a Medicaid card [14]. While several studies have shown a clear association between early prenatal care and better health outcomes for mothers and infants [19], others have also demonstrated that most women attend their first prenatal care appointment around nine weeks into gestation on average, with a two-weeks gap between when they confirm their pregnancy and when they attend their first prenatal clinic [14]. These are not too different from the findings in this study, where four weeks was the earliest gestational age at enrolment into prenatal care and 13.5 weeks the most common.

The pregnancy risks presented by the women in this study were categorised into four groups: Medicaid health insurance risks, medical complication risks, gestational age-related risks, and prenatal registration risks. The Medicaid risks are a complete lack of Medicaid health insurance or the wrong Medicaid coverage. In either case, the women could not be enrolled into managed care and could not secure a doctor's appointment for prenatal care. 53% (n = 32) of the women had no insurance at all, while 45% (n = 27) were on the wrong Medicaid insurance. Pregnancy Medicaid (TP 44) is the ideal Medicaid coverage and is required for these women to enrol in prenatal care. This total lack of appropriate health insurance for prenatal care is a risk factor for poor maternal and child outcomes, as mothers and babies may be denied care for serious health conditions. Several studies have demonstrated a positive link between comprehensive health insurance coverage for mothers and better postpartum visit attendance, as well as fewer emergency department visits for mothers and babies [20].

Among the medical complications seen with the women in this study, depression (8%), anxiety (5%), and seizure disorders (3%) were the most common. Teenage pregnancy accounted for 7% (n = 4) and advanced maternal or geriatric age accounted for 5% (n = 3). Teenage pregnancy is defined as pregnancy in women within the age bracket of 13 to 19 years, while 35 years and above is considered to be advanced maternal age.

Among women with lack of appropriate Medicaid insurance coverage, African-American women made up the largest proportion, at 51%, followed by Hispanic women, at 37%, and the White women at 12%. A similar distribution is seen with medical complication risks, where African American women accounted for 53%, Hispanic women accounted for 32%, and white women accounted for 16%. Hispanic women accounted for the greatest proportion of age-related risk and late prenatal registration risks, at 50% and 52% respectively. This is followed by African American, who accounted for 30% of gestational age risks and 45% of late prenatal registration risk. White women remained consistently at or below 20% in all the risk categories, especially the late prenatal registration risk group, where they accounted for about 3%. These differential risks reflect the health disparities among vulnerable populations of American women who are at highest risk for pregnancy-related mortality [21].

Concerning registration by MCOs, it took the women between four and 150 days to complete registration, with an average of 53 days. An MCO is an intermediary health care provider set up to manage health care costs, as well as improve health-service utilisation and quality of care by coordinating and overseeing the delivery of health services. State governments contract with various types of MCOs to deliver Medicaid health care services to beneficiaries and pay them per member per month for these services (capitation). The implication of this is that women need to enrol into MCO before they can secure a doctor's appointment for prenatal care. The nurse-led admission process at the New Life prenatal care allowed us to screen for risks, schedule and deliver initial doctor's appointments for high-risk clients who need urgent care earlier, booking appointments between one day to 59 days after registration by our organisation, with an average of 22 days. This shows the importance of a nurse-led managed care for pregnant women, where risk assessment is done at first contact with the client and appropriate referrals are made to doctors while waiting for enrolment by an MCO.

## 5. Conclusion

With the high prevalence of various pregnancy related risks among pregnant women in Houston, Texas, there is an urgent need for these women to be seen early in their first trimester by health care providers. One way to facilitate this is through the engagement of community health organisations such as the New Life Perinatal Health Care Services.

When such an organisation provides a nurse led care management of pregnancy, it can facilitate quality prenatal care by engaging in pregnancy risk assessment and making appropriate doctors' referrals. These Doctors referrals are made whether the women had appropriate Medicaid insurance or not.

These quick risk mitigation services go a long way in improving pregnancy outcomes, as well as reducing perinatal morbidity and mortality for mothers and children.

All 60 women who registered with our organisation were promptly screened for pregnancy-associated risks at first contact, and appropriate referrals were made for those who need to see a doctor as soon as possible. The outcome was that they all had deliveries of live babies and expressed very high satisfaction with the services offered to them [Figure 3].

### Recommendations

1. Enlarge the capacity of the presently engaged nurse-led community-based organisations in Houston, Texas to admit more pregnant women for enrolment into early prenatal care.
2. Engage new nurse-led community-based organisations to facilitate early prenatal care for women in Houston, Texas.
3. Replace the CPW program with Targeted case management services for pregnant women and infants.
4. Policy changes for the initiation of prenatal care are recommended for specific gestational weeks based on relevant health professionals' advice and input.
5. Expand insurance coverage to prioritise early prenatal care in high-risk populations.
6. Create and increase awareness of the importance of early prenatal care.
7. Enhance efforts to facilitate birth control access to reduce rates of unintended pregnancies, especially at extreme of age (teenagers and advanced age).
8. Innovation with technology-based prenatal care visits
9. Reduce the interval between admission to enrolment into case management from an average of 53 days to 14 days.
10. Reduce the interval between admission to first doctor's for high-risk cases from an average of 22 days to two days.
